# Paediatric Comment

**Published:** 1956-04

**Authors:** 


### PAEDIATRIC COMMENT

" To begyn a treatyse of the cure of children we should declare somewhat of the principles of ye generacion; they being in ye womb, the time of the procedinge, the maner of the birth, . . . for as muche as these things are very true and manifeste, some perteyning to the offyce of a midwyfe, others for the reverence of the matter . . . and I will employ my studyes to the honour of God and profit of the weale publike."

In these words Thomas Phaer (1545) shows no mean insight into Child Health. There is no narrow view in this and a notable exclusion of any crippling specialism. We have moved a long way from " the diseases of children " to a wider view contained in the phrase " sick children ", and then on to the global notions implied in " Child Health ".

Medical knowledge and skills advance; so also must wisdom and all those matters appertaining to the love, care, kindness, well-being and human example upon which the rising generation may grow and flourish in physical, emotional and spiritual health, and security. Such is medicine with a very big M. Many persons are involved in all this and perspectives must be kept in mind. The home, the school, the street, and sometimes the hospital—or even an institution, may all come into the picture. More and more depends upon the human relationships existing amongst those who are engaged in the various and many contributions which are adding their daily impact upon the healthy or the sick child. The child's mind, like the body, is very labile for good or for ill, especially in the early and vulnerable years. No conflict of interests should warp a happy natural growth and progress.

Immense amounts of time and effort are being directed towards protection and care in child life and health. In fact, every responsible member of a well-ordered community is making some direct or indirect contribution. Voluntary and statutory arrangements abound, and, we always hope, closely co-operate. Governments may enact, but real effectiveness depends upon each and all running as teams and revealing a complementary effectiveness. To strive for health and to have a lively awareness of the possibilities in nature and nurture is an aspect of the Health Service yet to be more fully developed. Natural endowment and the environment are major determinants which need keen and subtle assessment. Thus each and all who can contribute to the sum total of the good in the home and the family and the community should themselves work and think together. Thus, doctors, medical students, nurses, midwives, health visitors and members of other services should continue to strive for unity of purpose and be increasingly happy in the knowledge that the rising generation will reflect the goodness accordingly.

Professor W. S. Craig has enlightened us further on these matters, not only in his book *Child and Adolescent Life in Health and Disease*, but in an address printed in this issue.

